# Autophagy Inhibitors Do Not Restore Peroxisomal Functions in Cells With the Most Common Peroxisome Biogenesis Defect

**DOI:** 10.3389/fcell.2021.661298

**Published:** 2021-04-01

**Authors:** Femke C. C. Klouwer, Kim D. Falkenberg, Rob Ofman, Janet Koster, Démi van Gent, Sacha Ferdinandusse, Ronald J. A. Wanders, Hans R. Waterham

**Affiliations:** ^1^Laboratory Genetic Metabolic Diseases, Amsterdam Gastroenterology, Endocrinology and Metabolism, Amsterdam University Medical Centers – Location AMC, University of Amsterdam, Amsterdam, Netherlands; ^2^Department of Pediatric Neurology, Emma Children’s Hospital, Amsterdam University Medical Centers – Location AMC, University of Amsterdam, Amsterdam, Netherlands

**Keywords:** peroxisome biogenesis disorder, Zellweger spectrum disorder, autophagy inhibitors, pexophagy, chloroquine, hydroxychloroquine, L-arginine, peroxisomal functions

## Abstract

Peroxisome biogenesis disorders within the Zellweger spectrum (PBD-ZSDs) are most frequently associated with the c.2528G>A (p.G843D) mutation in the *PEX1* gene (PEX1-G843D), which results in impaired import of peroxisomal matrix proteins and, consequently, defective peroxisomal functions. A recent study suggested that treatment with autophagy inhibitors, in particular hydroxychloroquine, would be a potential therapeutic option for PBD-ZSD patients carrying the PEX1-G843D mutation. Here, we studied whether autophagy inhibition by chloroquine, hydroxychloroquine and 3-methyladenine indeed can improve peroxisomal functions in four different cell types with the PEX1-G843D mutation, including primary patient cells. Furthermore, we studied whether autophagy inhibition may be the mechanism underlying the previously reported improvement of peroxisomal functions by L-arginine in PEX1-G843D cells. In contrast to L-arginine, we observed no improvement but a worsening of peroxisomal metabolic functions and peroxisomal matrix protein import by the autophagy inhibitors, while genetic knock-down of ATG5 and NBR1 in primary patient cells resulted in only a minimal improvement. Our results do not support the use of autophagy inhibitors as potential treatment for PBD-ZSD patients, whereas L-arginine remains a therapeutically promising compound.

## Introduction

The Peroxisome Biogenesis Disorders (PBDs), which include the Zellweger spectrum disorders (PBD-ZSDs) and Rhizomelic Chondrodysplasia Punctata type 1, comprise a group of autosomal recessive metabolic disorders associated with multi-organ defects due to the loss of functional peroxisomes (Klouwer et al., 2015; Braverman et al., 2016). Peroxisomes are single-membrane-bound organelles present in virtually every human cell and involved in several crucial metabolic processes. These include, among others, the α- and β-oxidation of branched-chain and very long chain fatty acids (VLCFAs), the synthesis of bile acids and ether phospholipids, including plasmalogens, and the homeostasis of reactive oxygen species (Wanders and Waterham, 2006).

PBD-ZSDs can be caused by bi-allelic mutations in any of 12 different *PEX* genes, which encode PEX proteins, also known as peroxins, that are required for the import of peroxisomal matrix and/or membrane proteins. Defects in any of these PEX proteins result in a defective peroxisome biogenesis (assembly), which thus affects peroxisome-dependent metabolic processes. This results in characteristic metabolic aberrations reflecting peroxisomal dysfunction, including increased levels of VLCFAs, such as C26:0, pristanic acid and bile acid precursors, and decreased levels of plasmalogens, mature bile acids and docosahexaenoic acid (DHA) (Wanders and Waterham, 2006; Waterham et al., 2016).

Approximately 60% of PBD-ZSD patients have bi-allelic mutations in the *PEX1* gene of whom 40% are compound heterozygous or homozygous for the missense mutation c.2528G>A, which results in a p.G843D amino acid change causing impaired PEX1 protein function (Ebberink et al., 2011). PEX1 forms a heterohexamer with PEX6 to constitute the peroxisomal AAA ATPase complex, which is involved in the export of PEX5, the cytosolic receptor for PTS1-targeted peroxisomal matrix proteins, from the peroxisome back into the cytosol after delivery of its cargo (Miyata and Fujiki, 2005). A severe PEX1 defect completely impairs the import of peroxisomal matrix proteins resulting in reduced numbers of ‘empty’ peroxisomal vesicles, also known as ‘peroxisomal ghosts’, in PBD-ZSD cells (Santos et al., 1988). The *PEX1-c.2528G*>*A* mutation is associated with a more attenuated clinical presentation and often results in peroxisome mosaicism in cell populations, with some cells having normal appearing peroxisomes while other cells have peroxisomal ghosts (Imamura et al., 1998). In cells from patients with severe defects in PEX3, PEX16 and PEX19, however, no peroxisomal ghosts are observed, in keeping with their role in the import of peroxisomal membrane proteins (Waterham et al., 2016).

To date, there is no therapeutic treatment for PBD-ZSDs. In recent years, however, several small compounds have been identified, which improve peroxisomal matrix protein import and, importantly, peroxisomal functions in cultured cells of milder affected PBD-ZSD patients, including in particular patients carrying one or two *PEX1-c.2528G*>*A* alleles. Among these are 4-phenylbutyrate (Wei et al., 2000), betaine, trimethylamine N-oxide, glycerol (Zhang et al., 2010), L-arginine (Berendse et al., 2013), and diosmetin (MacLean et al., 2019). The mechanisms underlying the observed improvement of peroxisomal matrix protein import and function in the cells by these compounds are largely unknown, however.

A recent report by Law et al. (2017) claimed that inhibition of autophagy, including the autophagic degradation of peroxisomes also known as pexophagy, restores peroxisomal functions in PBD-ZSD cells harboring the PEX1-G843D mutation. In this study the PEX1/PEX6 complex-dependent export of PEX5 from peroxisomes was postulated to act as a peroxisome quality control system that prevents pexophagy. A defective PEX1/PEX6 complex was found to increase pexophagy and rapid degradation of peroxisomal ghosts, based on which it was postulated that the observed impaired peroxisomal matrix protein import is a consequence of increased pexophagy rather than a direct consequence of dysfunctional PEX1 or PEX6. The authors therefore hypothesized that inhibition of autophagy in cells with impaired PEX1/PEX6 complex functioning would increase the half-life of the peroxisomal ghosts, thereby increasing the possibility for PEX5 to import peroxisomal matrix proteins into these ghosts. If the case, this should then also lead to improved peroxisomal metabolic functions, which, however, had not been studied. The authors primarily focused on hydroxychloroquine (HCQ) to inhibit autophagy, as this compound is FDA approved for the treatment of malaria.

Based on earlier studies reporting that autophagy in cells can be inhibited by high concentrations of L-arginine (Xia et al., 2016) and induced when L-arginine is depleted (García-Navas et al., 2012; Angcajas et al., 2014), it was hypothesized (Nazarko, 2017) that pexophagy inhibition might also be the mechanism underlying the improved peroxisomal protein import and functions in PBD-ZSD cells harboring the PEX1-G843D mutation treated with L-arginine (Berendse et al., 2013). To investigate this hypothesis, we have here compared the positive effect of L-arginine on the import of peroxisomal matrix proteins and restoration of peroxisomal metabolic functions to the effect of the autophagy inhibitors chloroquine, hydroxychloroquine and 3-methyladenine in four different PEX1-G843D cell types, including primary fibroblast cells from PBD-ZSD patients. In contrast to L-arginine, which markedly improved peroxisomal functions, we found that the autophagy inhibitors did not improve endogenous peroxisomal matrix protein import or peroxisomal metabolic functions, but in fact caused a further decrease of the peroxisomal functions in all cell lines, including the primary patient cells. A stable genetic knock-down of NBR1 or ATG expression in primary patient cells using RNAi resulted in only a minimal improvement. Our results argue strongly against the use of autophagy inhibitors as potential therapy for PBD-ZSD patients.

## Results

### Effect of Compounds on Peroxisomal Functions in Primary Skin Fibroblasts of Patients

We previously showed that L-arginine improves peroxisomal matrix protein import and peroxisomal metabolic functions in cultured primary skin fibroblasts derived from PEX1-G843D patients (Berendse et al., 2013). To study if this is due to inhibition of autophagy by L-arginine in these cells as was recently suggested (Nazarko, 2017), we first compared the effect of L-arginine with the effects of the early autophagy inhibitor 3-methyladenine (3-MA), and two late autophagy inhibitors chloroquine (CQ) and hydroxychloroquine (HCQ) in primary skin fibroblasts derived from two different PEX1-G843D patients, a PEX1-null patient and a control individual. Cells were incubated for 7 days with the different compounds or, as a positive control, with glycerol, which was previously shown to efficiently restore peroxisomal protein import and functions in PEX1-G843D patient cells (Zhang et al., 2010; Berendse et al., 2013).

**FIGURE 1:**
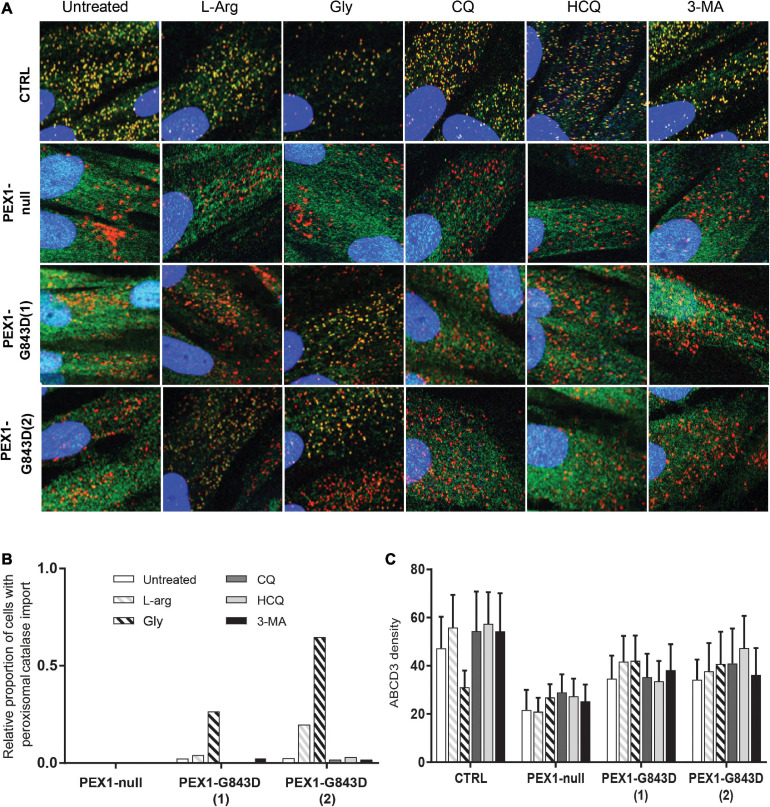
Autophagy inhibitors do not restore import of peroxisomal catalase in primary PEX1-G843D fibroblasts. **(A)** Immunofluorescence microscopy in primary fibroblasts from a control individual (CTRL), a PBD-ZSD patient without residual PEX1 function (PEX1-null) and two PBD-ZSD patients homozygous for the PEX1-G843D mutation (PEX1-G843D(1) and PEX1-G843D(2)). Cells were incubated with 20 mM L-arginine (L-Arg), 5% glycerol (Gly), 10 μM chloroquine (CQ), 10 μM hydroxychloroquine (HCQ) or 10 mM 3-methyladenine (3-MA) for 7 days, before determining the subcellular localization of the peroxisomal matrix protein catalase (green signal) and the peroxisomal membrane protein (PMP) ABCD3 (red signal). Shown are representative images, each image representing a section of 40 × 40 μm. The nuclei were stained with DAPI (blue signal). **(B)** Quantification of primary fibroblasts, treated as described in A, which display peroxisomal catalase import as determined by immunofluorescence microscopy (> 60 cells analyzed for each condition). Control fibroblasts showed 100% peroxisomal catalase import under all conditions (set as 1.0). **(C)** Density of punctated ABCD3-containing structures (indicating peroxisomal membranes) per cell area as determined by immunofluorescence microscopy in primary fibroblasts treated as described in A (see section “Materials and Methods” for more details). Data presented as mean of *n* > 40 cells + SD. Statistical analyses of treated versus untreated cells were performed using Mann-Whitney *U* tests and revealed no statistically significant differences.

We first investigated the effect of the compounds on peroxisomal import of the PTS1-targeted peroxisomal matrix protein catalase using immunofluorescence microscopy. In control cells, catalase was correctly localized to peroxisomes under all conditions, as indicated by its co-localization with the peroxisomal membrane protein ABCD3 (Figure 1A). In the vast majority of untreated PEX1-G843D cells, catalase was mislocalized to the cytosol. However, as previously reported (Berendse et al., 2013), the proportion of PEX1-G843D cells showing peroxisomal catalase import clearly increased when they were incubated with glycerol, and – be it to a lesser extent – with L-arginine (Figures 1A,B). In contrast, peroxisomal catalase import did not improve in cells incubated with any of the autophagy inhibitors (Figure 1B). In the PEX1-null cell line, which contains peroxisomal ghosts as confirmed by the punctated ABCD3 labeling, peroxisomal catalase import remained completely absent after incubation with any of the compounds, including L-arginine and glycerol (Figures 1A,B).

**FIGURE 2:**
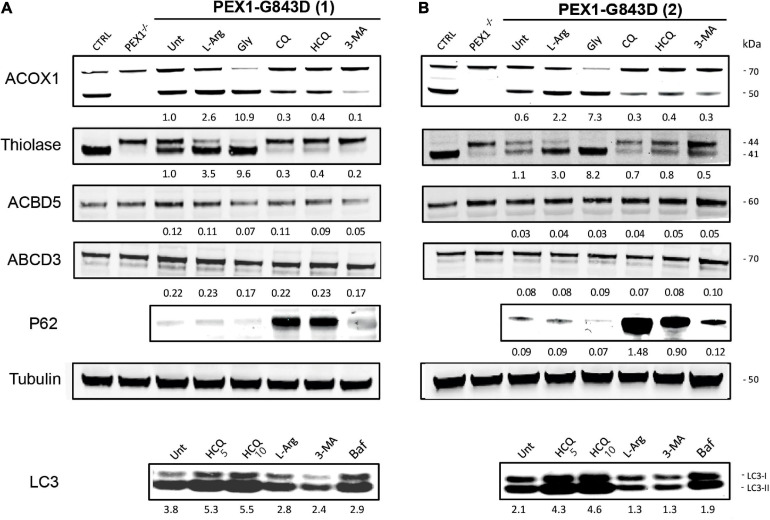
Autophagy inhibitors do not restore peroxisomal import of PTS1- and PTS2-targeted peroxisomal matrix proteins in primary PEX1-G843D fibroblasts. Immunoblot analysis of homogenates of primary fibroblasts from two PBD-ZSD patients homozygous for the PEX1-G843D mutation [PEX1-G843D(1) **(A)** and PEX1-G843D(2) **(B)**] treated as described in Figure 1A. The protein levels of the PMPs ACBD5 and ABCD3 and the ratio of the processed over unprocessed forms of PTS1-targeted ACOX1 and PTS2- targeted thiolase were determined using specific antibodies. Below the ACOX1 bands, the corresponding ratios between the intensity of processed (50 kDa) over unprocessed (70 kDa) ACOX1 bands are indicated. Below the thiolase bands, the corresponding ratios between the intensity of processed (41 kDa) over unprocessed (44 kDa) thiolase bands are indicated. Below the ACBD5 and ABCD3 bands, the corresponding signal intensity relative to the tubulin band intensity, which was used as a loading control, is indicated. Homogenates of non-treated control fibroblasts and PEX1-null fibroblasts (PEX1^–/–^) were used for reference. The effect of the different treatments on autophagy was assessed by determining the relative protein levels of the autophagy adaptor protein SQSTM1/P62 (P62; relative to tubulin levels) and the ratio of MAPILC3B-II (LC3-II) over MAPILC3B-I (LC3-I). Cells were incubated for 7 days with the different compounds, except for bafilomycin (24 h incubation). Experiments were repeated twice; shown are representative blots from one experiment.

To confirm that the treatment of the different autophagy inhibitors indeed resulted in inhibition of autophagy in the PEX1-G843D cells, we determined their effect on the levels of the autophagy adaptor protein SQSTM1/P62 (P62) and on the levels and conversion of MAPILC3B-I (LC3-I) to MAPILC3B-II (LC3-II) using immunoblot analysis. Treatment with CQ and HCQ resulted in a marked increase in P62 levels, while 3-MA treatment caused a modest increase. In contrast, treatment with L-arginine and glycerol did not result in a change of P62 levels when compared to untreated cells (Figure 2). HCQ treatment resulted in increased levels and conversion of LC3-I to LC3-II, while bafilomycin treatment resulted in increased LC3-1 levels. Treatment with 3-MA resulted in lower levels of both LC3-I and LC3-II and a decreased conversion of LC3-I to LC3-II, when compared to untreated cells. Treatment with L-arginine also resulted in lower LC3-I and LC3-II levels and a decreased conversion (Figure 2).

Next, we assessed functional peroxisomal matrix protein import in the cells treated with the different compounds by examining the processing of the PTS1-targeted peroxisomal matrix protein acyl-CoA oxidase 1 (ACOX1) and the PTS2-targeted matrix protein thiolase. Both proteins are synthesized in the cytosol as larger precursor proteins and only processed into their smaller mature forms after import into peroxisomes, making their processing efficiency a sensitive read-out for functional peroxisomal matrix protein import in cells (Schram et al., 1986; Berendse et al., 2013).

ACOX1 is synthesized as a 70kDa precursor protein, which only inside peroxisomes can be processed into a 50kDa and a 20kDa protein. Although in control cells the intra-peroxisomal processing of ACOX1 is not always 100%, its processing is completely absent in PEX1-null cells (Figure 2). Compared to non-treated PEX1-G843D cells, ACOX1 processing is markedly increased when these cells were incubated with glycerol and, albeit less, L-arginine. Incubations of the cells with CQ, HCQ and 3-MA, however, did not increase but decrease ACOX1 processing (Figure 2).

Processing of the 44kDa precursor of the PTS2-targeted thiolase into its 41kDa mature form is complete in the control cells, but fully blocked in the PEX1-null cells (Figure 2). In untreated PEX1-G843D cells, we observed equal amounts of processed and unprocessed thiolase (Figure 2). In keeping with what we observed for catalase and ACOX1 import, thiolase processing clearly increased when the PEX1-G843D cells were incubated with glycerol and L-arginine, but decreased when the cells were incubated with CQ, HCQ and 3-MA (Figure 2).

**FIGURE 3:**
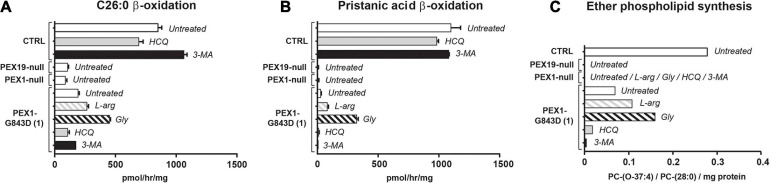
Autophagy inhibitors do not restore peroxisomal metabolic functions in primary PEX1-G843D fibroblasts. Peroxisomal β-oxidation activity of C26:0 **(A)** and pristanic acid **(B)**, and **(C)** *de novo* synthesis of the ether phospholipid PC-(O-37:4) determined in primary fibroblasts from a PBD-ZSD patient homozygous for the PEX1-G843D mutation (PEX1-G843D(1)) and a control individual (CTRL) treated as described in Figure 1A. For reference we included the peroxisomal β-oxidation activities and the *de novo* PC-(O-37:4) synthesis values measured in untreated primary fibroblasts from a PBD-ZSD patient without residual PEX1 function (PEX1-null; still containing peroxisomal membranes) and from a PBD-ZSD patient without residual PEX19 function (PEX19-null; lacking peroxisomes entirely). β-oxidation activities are expressed as pmol/hr/mg protein and as mean + SD (*n* = 3); ether phospholipid synthesis is expressed as PC-(O-37:4) over PC-(28:0) over mg protein (mean of two measurements); PC-(28:0) was used as internal standard.

To assess the effect of the different compounds on peroxisomal metabolic processes, we measured the peroxisomal β-oxidation activity of C26:0 and pristanic acid, and the peroxisome-dependent *de novo* synthesis of ether phospholipids in treated PEX1-G843D and control fibroblasts (Figure 3). Whereas the β-oxidation activity for both C26:0 and pristanic acid was clearly decreased in untreated PEX1-G843D cells when compared to the control cells, PEX1-G843D cells incubated with L-arginine and, in particular, glycerol showed a clear increase in β-oxidation activity (Figures 3A,B). In contrast, incubation of these cells with the autophagy inhibitors HCQ and 3-MA had a negative effect on the β-oxidation of both fatty acids. The peroxisome-dependent *de novo* synthesis of the ether phospholipid PC-(O-37:4) was measured following incubation of cells with 1-heptadecanol (C17:0-OH) (see section “Materials and Methods”). PC-(O-37:4) synthesis was decreased in untreated PEX1-G843D cells when compared to the control cells, but increased when the PEX1-G843D cells were incubated with L-arginine and glycerol (Figure 3C). In contrast, incubation with HCQ and 3-MA caused a further decrease of PC-(O-37:4) synthesis in PEX1-G843D cells.

Taken together, our results show that while L-arginine and glycerol clearly improve peroxisomal functions, there is no positive but even a negative effect of the autophagy inhibitors HCQ and 3-MA on peroxisomal functions and peroxisomal matrix protein import in primary patient cells. Thus, our findings do not support the claim by Law et al. (2017) that autophagy inhibitors, in particular HCQ, restore peroxisomal functions in PBD-ZSD cells harboring the PEX1-G843D mutation. However, because Law et al. (2017) used the levels of peroxisomal membrane proteins (PMPs) and in particular the abundance of ABCD3-positive peroxisomal membranes (‘ghosts’) as primary readout, we also determined these parameters in the primary patient cells. We did not find significant changes in the protein levels of the PMP ACBD3 and a second abundant PMP ABCD5 (Figure 2) nor did we find an increased density of ABCD3-positive peroxisomal membranes (Figure 1C) in PEX1-G843D or PEX1-null cells upon incubation with any of the tested compounds.

### Effect of Compounds on Peroxisomal Functions in Non-primary PEX1-G843D Cells

To determine if the effect of autophagy inhibitors on peroxisomal functions varies among different cell types, we repeated our studies with the exact same transformed and immortalized PEX1-G843D fibroblast cell line (tr/immPEX1-G843D) used by Law et al. (2017).

**FIGURE 4:**
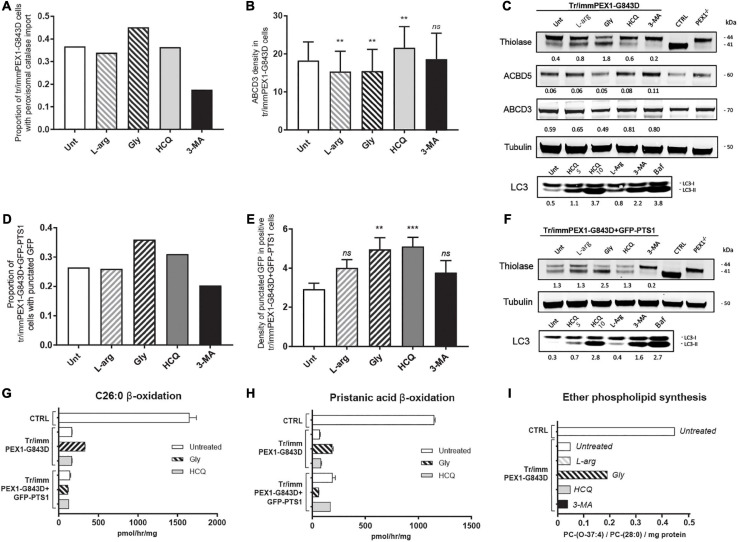
Autophagy inhibitors do not restore import of peroxisomal matrix proteins and metabolic functions in transformed-and-immortalized patient cells. The effect of treatment with different compounds on peroxisomal matrix protein import and peroxisomal metabolic functions was assessed in previously reported PEX1-G843D fibroblasts that had been transformed and immortalized (tr/immPEX1-G843D) (Zhang et al., 2010) as well as in the same cells stably overexpressing GFP-PTS1 (tr/immPEX1-G843D + GFP-PTS1) (Zhang et al., 2010). Cells were treated with 20 mM L-arginine (L-arg), 5% glycerol (Gly), 10 μM hydroxychloroquine (HCQ), or 10 mM 3-methyladenine (3-MA) for 24 h. **(A)** Quantification of tr/immPEX1-G843D cells that display functional peroxisomal catalase import after treatment. Cells were analyzed by immunofluorescence microscopy to determine the subcellular localization of catalase (> 150 cells analyzed for each condition). **(B)** Density of ABCD3 punctated structures (indicating peroxisomal membranes) per cell area determined in treated tr/immPEX1-G843D cells using immunofluorescence microscopy (see Materials and Methods for more details). Data is presented as mean of *n* > 45 cells ± SD. **(C)** Immunoblot analysis of homogenates of tr/immPEX1-G843D cells, which were treated for 7 days with the different compounds. The protein levels and processing of thiolase and the PMPs ACBD5 and ABCD3 were determined using specific antibodies. Below the thiolase bands, the corresponding ratios between the intensity of processed (41 kDa) over unprocessed (44 kDa) thiolase are indicated. Below each ACBD5 and ABCD3 band the corresponding signal intensity relative to the tubulin band intensity, which was used as a loading control, is indicated. Homogenates of control (CTRL) and PEX1-null (PEX1^–/–^) primary fibroblasts were used as controls. To study the effect of different treatments on autophagy we determined the ratio of LC3-II over LC3-I (7 days incubation). For this, we also treated cells with 5 μM hydroxychloroquine and 10 μM bafilomycine (24 h). **(D)** Quantification of tr/immPEX1-G843D + GFP-PTS1 cells that display punctated GFP structures after treatment. Cells were analyzed by fluorescence microscopy to determine the subcellular localization of GFP (> 120 cells analyzed for each condition). **(E)** Density of punctated GFP structures per cell area in treated tr/immPEX1-G843D + GFP-PTS1 cells, which display GFP punctated structures (i.e., cells with cytosolic GFP excluded; see Materials and Methods for more details). Data is presented as mean of *n* > 40 cells ± SEM. **(F)** Immunoblot analysis of homogenates of tr/immPEX1-G843D + GFP-PTS1 cells, which were incubated for 7 days with the different compounds. See also at C for annotations. **(G,H)** Peroxisomal β-oxidation activity of C26:0 **(G)** and pristanic acid **(H)** in tr/immPEX1-G843D and tr/immPEX1-G843D + GFP-PTS1 cells, which were incubated for 7 days with 5% glycerol (Gly) or 10 μM hydroxychloroquine (HCQ). Primary fibroblasts derived from a control individual were used as reference. The activity is presented in pmol/hr/mg protein as mean + SD (*n* = 3). **(I)** *de novo* synthesis of the ether phospholipid PC-(O-37:4) in tr/immPEX1-G843D cells which were treated for 7 days with the different compounds. Primary fibroblasts derived from a control individual were used as reference. Ether phospholipid synthesis is expressed as PC-(O-37:4) over PC-(28:0) over mg protein (mean of two measurements); PC-(28:0) was used as internal standard. Statistical analyses of treated cells versus untreated cells in panels **(B,E)** were performed using Mann Whitney *U* tests (**p < 0.01, ***p < 0.001, *ns* not significant).

Using the same incubation time as Law et al. (2017), 24 h, we observed an increase in catalase import in tr/immPEX1-G843D cells treated with glycerol but no increase in the cells treated with HCQ or L-arginine and even a decrease in the cells treated with 3-MA (Figure 4A). In contrast to our results with the primary cells, but similar as reported by Law et al. (2017), we observed a positive effect of HCQ on the density of ABCD3-positive peroxisomal membrane vesicles in this cell line (Figure 4B). Also the protein levels of the PMPs ACBD3 and ABCD5 (Figure 4C) increased slightly after incubating the cells for 7 days with HCQ and 3-MA. Importantly, however, the treatment with HCQ and 3-MA caused a decrease in thiolase levels and, in the case of 3-MA, thiolase processing (Figure 4C), in contrast to the treatment with glycerol and L-arginine which improved the processing.

Similar as for the primary cells, we observed a small increase in the levels and conversion of LC3-I to LC3-II in the L-arginine-treated tr/immPEX1-G843D cells when compared to untreated cells, and a strong increase upon incubation with HCQ and bafilomycin (Figure 4C). In contrast to what we observed in the primary cells, we noted that 3-MA treatment also resulted in increased levels and an increased conversion of LC3-I to LC3-II when compared to untreated cells (Figure 4C).

Law et al. (2017) also reported improved peroxisomal import by HCQ based on the observation that tr/immPEX1-G843D fibroblasts which stably overexpress GFP-PTS1, i.e., green fluorescent protein reporter with a peroxisomal matrix targeting signal, displayed more punctated GFP structures when treated with HCQ. Using the same cell line, tr/immPEX1-G843D + GFP-PTS1, we indeed also observed an increased proportion of cells with punctated GFP structures when incubated with HCQ and glycerol, but not with L-arginine and again a decrease with 3-MA (Figure 4D). In addition, the density of punctated GFP structures per cell increased upon incubation with HCQ, glycerol, L-arginine and 3-MA (Figure 4E), which indicates an increased import of GFP-PTS1 into peroxisomes. However, when we examined the processing of endogenous thiolase, we only observed an increase when these cells were incubated with glycerol (Figure 4F), implying that the reporter protein GFP-PTS1 behaves different than endogenous peroxisomal proteins.

The effect of the different compounds on the levels and conversion of LC3-I to LC3-II in the tr/immPEX1-G843D + GFP-PTS1 cells was similar to the effects observed in the tr/immPEX1-G843D cells with again an increase of these upon the treatment with 3-MA.

As for the primary cells, we also measured the peroxisomal β-oxidation activity of C26:0 and pristanic acid to assess the effect of HCQ and glycerol on peroxisomal metabolic functioning in these two cell lines. We observed only some increased β-oxidation activity in the tr/immPEX1-G843D cells when treated with glycerol, but no increase with HCQ in both cell lines, or with glycerol in the tr/immPEX1-G843D + GFP-PTS1 cells (Figures 4G,H). The peroxisome-dependent *de novo* synthesis of the ether phospholipid PC-(O-37:4) was increased only when the tr/immPEX1-G843D cells were treated with glycerol, but remained low when these cells were treated with HCQ, 3-MA or L-arginine (Figure 4I).

**FIGURE 5:**
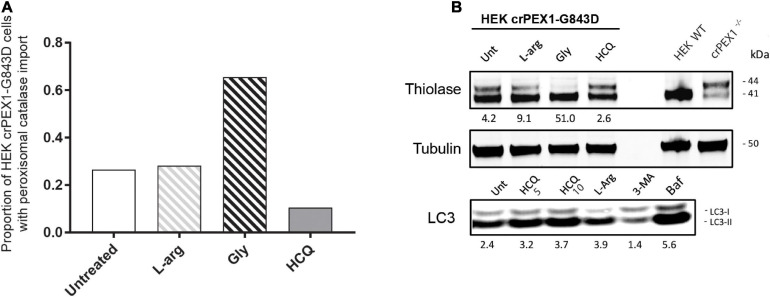
Autophagy inhibitors do not restore import of peroxisomal matrix proteins in HEK 293 cells harboring the PEX1-G843D mutation. The effect of treatment with different compounds on peroxisomal matrix protein import was assessed in HEK 293 cells, which are functionally homozygous for PEX1-G843D introduced by Crispr-Cas9 genome editing. The HEK crPEX1-G843D cells were incubated with 20 mM L-arginine (L-arg), 2.5% glycerol (Gly) or 10 μM hydroxychloroquine (HCQ) for 4 days. Since 3-MA incubation was found to be lethal for the HEK crPEX1-G843D cells, we excluded this treatment from the experiments. Wild-type HEK 293 cells (HEK WT) and Crispr-Cas9-generated HEK 293 cells with no functional PEX1 (crPEX1^–/–^) were used for reference. **(A)** Quantification of HEK crPEX1-G843D cells that display functional peroxisomal catalase import after treatment. Cells were analyzed by immunofluorescence microscopy to determine the subcellular localization of catalase (> 300 cells analyzed for each condition). **(B)** Immunoblot analysis of homogenates of treated HEK crPEX1-G843D cells. The processing of thiolase was determined using specific antibodies. Below the thiolase bands, the corresponding ratios between the intensity of processed (41 kDa) over unprocessed (44 kDa) thiolase are indicated. To study the effect of different treatments on autophagy we determined the ratio of LC3-II over LC3-I. For this, we also treated cells with 5 μM hydroxychloroquine and 10 μM bafilomycine.

Next, we studied the effect of L-arginine, HCQ and glycerol in HEK 293 Flp-In cells in which we introduced the PEX1-G843D-causing missense mutation c.2528G>A in the genome by CRISPR-Cas9 genome editing. Similar as the patient-derived PEX1-G843D fibroblasts, untreated HEK crPEX1-G843D cells showed peroxisome mosaicism, with cells having normal appearing peroxisomes while other cells have peroxisomal ghosts with catalase mislocalized to the cytosol; thiolase is present in both its processed mature and unprocessed precursor form (Figures 5A,B). Incubation of the HEK crPEX1-G843D cells with glycerol for 4 days increased the proportion of cells with peroxisomal catalase import two-fold and thiolase processing was restored almost completely (Figures 5A,B). Although after 4 days of incubation with L-arginine, the proportion of cells with peroxisomal catalase import did not increase, there was a clear increase in thiolase processing. In contrast, incubation of the cells with HCQ resulted in a decrease in both catalase import (Figure 5A) and thiolase processing (Figure 5B).

### Effect of Compounds on Cellular VLCFA Levels

Law et al. (2017) used the decrease of C26:0-lysophosphatidylcholine (C26:0-lysoPC) levels in patient fibroblasts incubated with HCQ as a main indicator for improved peroxisomal functioning. The rationale behind this is that VLCFAs, such as C26:0, and VLCFA-derived species, such as C26:0-lysoPC or C26:0-carnitine, typically accumulate in blood and cells of PBD-ZSD patients (Klouwer et al., 2017), since VLCFAs require functional peroxisomes for their degradation by β-oxidation.

**FIGURE 6:**
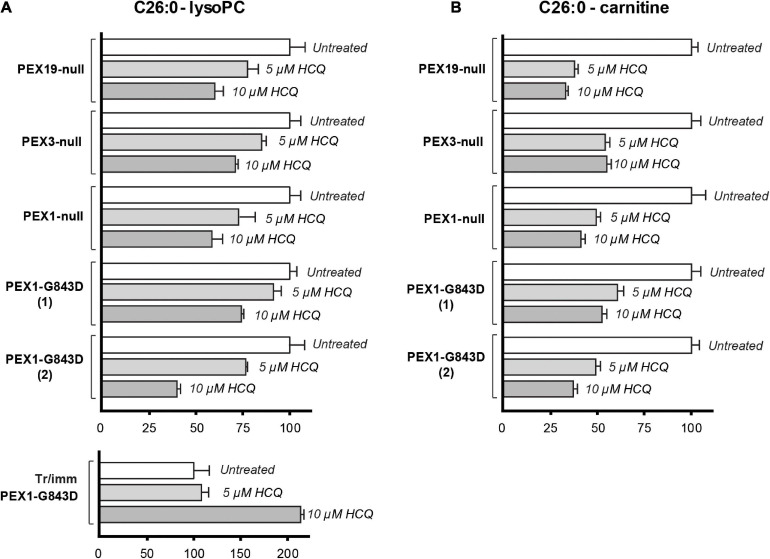
Effect of HCQ on cellular levels of VLCFAs. C26:0-lysophosphatidylcholine (C26:0-lysoPC) levels **(A)** and C26:0-carnitine levels **(B)** measured in untreated primary PEX1-null fibroblasts, two primary PEX1-G843D fibroblasts, primary PEX3-null fibroblasts, primary PEX19-null fibroblasts, tr/immPEX1-G843D cells and in the same cells after incubation with 5 or 10 μM hydroxychloroquine (HCQ) for 7 days. Levels in the treated cells are expressed as percentage of the mean of the levels in the corresponding untreated cells (set as 100%). Experiments were performed in duplicate; error bars represent SD.

Because our results so far did not reveal a restoration of peroxisomal metabolic functions upon incubation with HCQ, we repeated these experiments and analyzed the effect of HCQ on the levels of C26:0-lysoPC and C26:0-carnitine using primary skin fibroblasts from patients with different *PEX* gene defects. In addition to the primary PEX1-G843D and PEX1-null patient fibroblasts we used in this study, we included primary patient fibroblasts completely lacking peroxisomal ghosts due to the absence of PEX3 or PEX19 protein. We observed a marked decrease in the levels of C26:0-lysoPC and C26:0-carnitine in all primary fibroblasts treated with HCQ. The fact that this decrease occurred not only in the PEX1-G843D cells but also in the PEX1-null cells and even in the PEX3- and PEX19-deficient cells, implies that this effect by HCQ is not related to a restoration of peroxisomal functions by HCQ (Figure 6). Remarkably, when we analyzed the effect of HCQ on the levels of C26:0-lysoPC in the tr/immPEX1-G843D fibroblasts we observed an increase of the levels.

### Effect of Genetic Depletion of ATG5 and NBR1 on Peroxisomal Functions in Primary Skin Fibroblasts of Patients

The major aim of our study was to evaluate whether autophagy inhibitors, in particular HCQ, could restore peroxisomal functions in PEX1-G843D cells as previously claimed. Because such inhibitors may have non-specific effects unrelated to autophagy inhibition, however, we also studied whether autophagy inhibition by means of genetic depletion of selected autophagy genes could restore peroxisomal functions in the PEX1-G843D cells. To this end, we generated primary PEX1-G843D and control fibroblasts that stably express shRNAs against ATG5 or NBR1 and studied the effect of depleting ATG5 or NBR1 levels on peroxisomal matrix protein import and peroxisomal functions.

**FIGURE 7:**
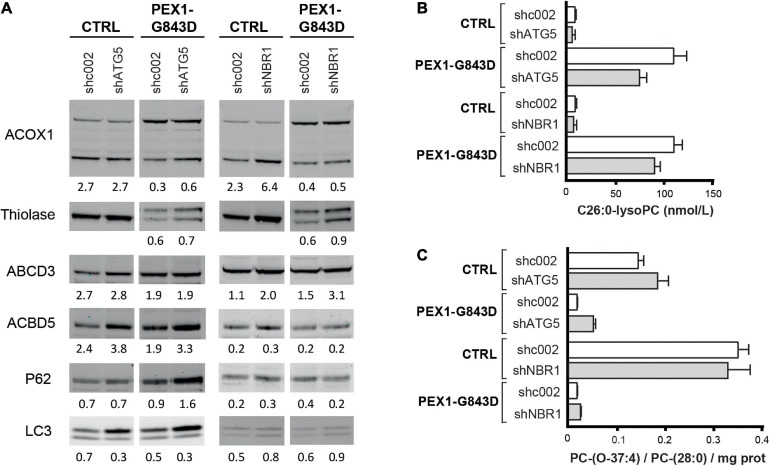
Effect of depletion of ATG5 or NBR1 in primary PEX1-G843D fibroblasts on peroxisomal matrix protein import and peroxisomal metabolic functions. **(A)** Immunoblot analysis of homogenates of primary PEX1-G843D fibroblasts and control fibroblasts that stably express shRNAs against ATG5 (shATG5) or NBR1 (shNBR1) or, as control, a non-specific scrambled shRNA (shc002). Cells were treated with the different compounds as described in Figure 1A. The relative protein levels of ACBD3, ABCD5 and P62 (corrected for tubulin or actin levels), the ratios of LC3-II over LC3-I and the ratios of the processed over unprocessed forms of PTS1-targeted ACOX1 and PTS2- targeted thiolase were determined as described in the legends of Figure 2. **(B)** C26:0-lysophosphatidylcholine (C26:0-lysoPC) levels measured in primary PEX1-G843D fibroblasts and control fibroblasts that stably express shRNAs against ATG5 (shATG5) or NBR1 (shNBR1) or a non-specific scrambled shRNA (shc002). **(C)** *De novo* synthesis of the ether phospholipid PC-(O-37:4) in primary PEX1-G843D fibroblasts and control fibroblasts that stably express shRNAs against ATG5 (shATG5) or NBR1 (shNBR1) or a non-specific scrambled shRNA (shc002) (expressed as PC-(O-37:4) over PC-(28:0) over mg protein; PC-(28:0) used as internal standard). Experiments were repeated twice; shown are representative blots from one experiment **(A)** and the means **(B,C)**.

ATG5 functions early in the autophagy signaling pathway and is involved in the elongation of the phagophore, its maturation into the complete autophagosome and in the conversion of LC3-I to LC3-II (Ye et al., 2018). Stable expression of shRNA-ATG5 in the control and PEX1-G843D cells resulted in a 70% reduction of ATG5 protein when compared to control and PEX1-G843D cells expressing a scrambled shRNA-002 (not shown). Upon ATG5 depletion, the P62 levels increased in the PEX1-G843D cells but remained similar in control cells (Figure 7A), while the LC3-I levels increased and the LC3-II levels remained unchanged in both the PEX1-G843D and control cells. ATG5 depletion in the PEX1-G843D cells resulted in a small increase in the processing of ACOX1 and thiolase, and of the levels of ACBD5, while the levels of ABCD3 remained similar (Figure 7A). Finally, the ATG5 depletion resulted in a slight decrease in C26:0-lysoPC levels (Figure 7B) and a slight increase in peroxisome-dependent *de novo* synthesis of the ether phospholipid PC-(O-37:4) in the PEX1-G843D cells (Figure 7C).

NBR1 acts as an autophagy receptor, which contains LC3- and ubiquitin-binding domains and mediates the targeting of polyubiquitinated aggregates to nascent autophagosomes, where it works cooperatively with p62 (Kirkin et al., 2009; Lamark et al., 2009). Previous work has identified NBR1 as the main autophagy receptor involved in pexophagy (Deosaran et al., 2013). Stable expression of shRNA-NBR1 in the control and PEX1-G843D cells resulted in a 85% reduction of NBR1 mRNA (not shown; NBR1 protein could not be detected with our antibodies in any of the cells) when compared to control and PEX1-G843D cells expressing a scrambled shRNA-002. NBR1 depletion had a slight effect on the P62 levels in the PEX1-G843D and control cells (Figure 7A), and resulted in a small increase in LC3 levels and LC3-I to LC3-II conversion. Similar as observed for ATG5 depletion, NBR1 depletion in the PEX1-G843D cells resulted in a small increase in the processing of ACOX1 and thiolase, while the levels of ACBD3 were increased, but the levels of ABCD5 remained similar (Figure 7A). NBR1 depletion also resulted in a slight decrease in C26:0-lysoPC levels (Figure 7B) and a slight increase in peroxisome-dependent *de novo* synthesis of the ether phospholipid PC-(O-37:4) in the PEX1-G843D cells (Figure 7C).

Thus, in contrast to treatment with autophagy inhibitors which resulted in a further decrease of peroxisomal functions in primary PEX1-G843D cells, a targeted genetic inhibition of pexophagy resulted in a slight improvement thereof.

## Discussion

PBD-ZSDs are severe metabolic disorders for which currently no therapeutic options are available. Yet, in recent years, cell-based screening assays identified several chemical compounds that have a positive effect on peroxisomal matrix protein import and peroxisomal functions in cells of milder affected patients carrying the PEX1-G843D mutation, and which thus may provide starting points for the development of therapy for such patients (Zhang et al., 2010; Berendse et al., 2013; MacLean et al., 2019). The mechanism by which the identified compounds improve peroxisomal protein import and functions have remained unknown but are crucial to resolve in guiding the development of targeted treatment strategies for PBD-ZSD.

A recent report claimed that inhibition of pexophagy with autophagy inhibitors may improve peroxisomal functions in PBD-ZSD cells harboring the PEX1-G843D mutation (Law et al., 2017). Based on their results, the authors patented the use of any potential autophagy-inhibiting compound, including the FDA-approved compound HCQ, as a potential treatment for PBD-ZS (patent WO 2015192198 A1). Unfortunately, the study did not study actual peroxisomal functions, which should have been a requirement for such a claim.

We initiated our study because L-arginine, which we previously showed to improve peroxisomal functions in cells of PBD-ZSD patients (Berendse et al., 2013), was also suggested to function as an autophagy inhibitor (Nazarko, 2017). We thus set out to compare the effect of L-arginine on restoring peroxisomal functions (Berendse et al., 2013) to the effect of different autophagy inhibitors, including HCQ, CQ and 3-MA, in different PEX1-G843D cell lines using multiple functional assays. These include assessment of the intra-peroxisomal processing of PTS1-targeted ACOX1 and PTS2-targeted thiolase, the peroxisomal β-oxidation activity of C26:0 and pristanic acid, the peroxisome-dependent *de novo* synthesis of ether phospholipids, and the cellular levels of C26:0-lysoPC. Notably, whereas treatment with L-arginine and the positive control glycerol (Zhang et al., 2010; Berendse et al., 2013) both clearly improved peroxisomal matrix protein import and peroxisomal metabolic functions, we did not observe a positive effect in any of the PEX1-G843D cell lines after incubation with the different autophagy inhibitors. In contrast, most functional assays showed that the autophagy inhibitors caused a worsening of these peroxisomal functions in these cells. This is probably due to non-specific effects of the inhibitors, because we observed that a genetic depletion of ATG5 and NBR1 resulted in a minor improvement in peroxisomal matrix protein import and functions in primary PEX1-G843D cells. These latter results indicate that inhibition of pexophagy in cells with residual peroxisomal functions may slightly improve these functions, but this is far less efficient than most of the chemical compounds that have been identified (Zhang et al., 2010; Berendse et al., 2013; MacLean et al., 2019).

Thus, our functional data do not confirm the conclusions drawn by Law et al. (2017), which were mainly based on indirect read-outs that are not necessarily an indicator for peroxisomal function. Indeed, similar as Law et al. (2017), we observed that the autophagy inhibitors resulted in an increased abundance of peroxisomal membrane proteins in some cell lines, including the cells used in their study. Importantly, however, these cells did not show improvement in peroxisomal functioning. Moreover, we found that the decrease in cellular C26:0-lysoPC levels upon treatment with HCQ not only occurred in PEX1-G843D cells, but also in PEX1-null cells, which completely lack functional peroxisomes but still have peroxisomal ghosts, and PEX3-null and PEX19-null cells, which even lack any peroxisomal structures, and thus is not due to a restoration of peroxisomal functions. Why HCQ treatment results in lowering of VLCFA/C26:0-lysoPC in the different PEX-null cells is unknown, but it is known that HCQ can have different effects on cellular lipid metabolism (Matsuzawa and Hostetler, 1980; Goldstein et al., 1975; Harder et al., 1980; Hostetler et al., 1985). It should be stressed here also that elevated VLCFA levels are only one of multiple biochemical aberrations occurring in cells of PBD-ZSD patients due to the lack of functional peroxisomes (Wanders and Waterham, 2006; Klouwer et al., 2015; Braverman et al., 2016; Waterham et al., 2016).

Similar as Law et al. (2017), we also noted an HCQ-induced increase in punctated GFP structures in the transformed and immortalized PEX1-G843D cells overexpressing GFP-PTS1, which showed co-localization with ABCD3. However, this should not be used as a single read-out for improved peroxisomal functioning, because all our additional functional assays showed that HCQ neither improved the peroxisomal import of endogenous matrix proteins nor peroxisomal β-oxidation activity or *de novo* ether phospholipid synthesis in these cells. Also, while the punctated GFP structures increased upon HCQ incubation, they decreased upon incubation with 3-MA. Moreover, in contrast to the primary patient fibroblasts, the tr/immPEX1-G843D cells showed a further increase of C26:0-lysoPC levels upon treatment with HCQ. The punctated GFP structures might be due to GFP accumulation in vesicles (Goodall et al., 2014), an increased targeting efficiency of GFP-PTS1 to malfunctioning peroxisomes that are not degraded as suggested by Law et al. (2017), or represent an overexpression artifact (Lisenbee et al., 2003; Voeltz et al., 2006; Rizzo et al., 2009; Delfosse et al., 2016).

## Conclusion

In conclusion, our study stresses the importance of using multiple, complimentary functional assays and cell lines when assessing the effect of chemical compounds on peroxisome functions in PBD-ZSD cells prior to considering clinical use. Moreover, our functional data do not support the use of autophagy inhibitors, including (H)CQ, for therapeutic treatment of PBD-ZSD patients. In contrast, we noted a worsening rather than an improvement of peroxisomal functions when using such inhibitors, which, in addition to the potential severe side effects of (H)CQ which recently regained attention due their potential use in the treatment of COVID-19 patients (Estes et al., 1987; Browning, 2002; Goldman et al., 2020; Tleyjeh et al., 2020), argues against the use thereof.

Finally, and in contrast to autophagy inhibitors, both L-arginine and glycerol clearly had a positive effect on peroxisomal functions in PBD-ZSD cells, which is unrelated to autophagy inhibition. Thus, these compounds and the resolution of their underlying mechanisms of action remain promising targets for future treatment options for PBD-ZSD patients.

## Materials and Methods

### Cell Culturing

For this study we used primary skin fibroblasts derived from PBD-ZSD patients homozygous for *PEX1*-c.2528G>A (PEX1-G843D), *PEX1*-c.2097dup (PEX1-null), PEX3-c.328_331del (PEX3-null), *PEX19*-c.763_764insA (PEX19-null) (Ebberink et al., 2011) transformed and immortalized skin fibroblasts compound heterozygous for the *PEX1*-c.2528G>A (p.G843D) and *PEX1*-c.2097dup (tr/immPEX1-G843D) (Zhang et al., 2010), tr/immPEX1-G843D cells overexpressing GFP-PTS1 (tr/immPEX1-G843D + GFP-PTS1) (Zhang et al., 2010), and HEK-293 Flp-In cells compound heterozygous for *PEX1*-c.2528G>A (p.G843D) and *PEX1*-c.2538_2541dup (HEK crPEX1-G843D) introduced by CRISPR-Cas9 genome editing (see below).

Primary skin fibroblasts were obtained according to standard procedures. Identifiable clinical and personal data from the patients were not available for this study. The previously reported transformed and immortalized PEX1-G843D/c.2097dup skin fibroblasts (named in this study tr/immPEX1-G843D and tr/immPEX1-G843D + GFP-PTS1) were a kind gift from dr. N.E. Braverman from McGill University Health Centre, Montreal, Canada, and are the same cell lines used in the study of Law et al. (2017). HEK 293 Flp-In cells used for CRISPR-Cas9 genome editing were obtained from Thermo Fisher, Waltham, MA, United States (R75007). All cell lines were cultured at 37°C under an atmosphere of 5% CO_2_ in Dulbecco’s modified Eagle’s medium (DMEM) supplemented with L-glutamine (BioWhittaker, Basel, Switzerland), 10% fetal bovine serum (Thermo Fisher, Waltham, MA, United States), 25 mM HEPES buffer (BioWhittaker Basel, Switzerland), 100 U/mL penicillin (Thermo Fisher, Waltham, MA, United States), 100 μg/mL streptomycin (Thermo Fisher, Waltham, MA, United States) and 250 ng/mL Fungizone (Thermo Fisher, Waltham, MA, United States).

Cells were incubated with 20 mM L-arginine (Sigma A5131), 5% glycerol (Acros Organics 158920025), 10 μM chloroquine (Sigma C6628), 5 or 10 μM hydroxychloroquine (Sigma H0915), 10 mM 3-methyladenine (Sigma M9281), or 10 μM bafilomycin as a positive control for autophagy inhibition. The compounds were dissolved in the culture medium, which was then filtered over a 0.45 μm Millex syringe filter (Merck, Darmstadt, Germany) for sterilization. One day after seeding the cells, the culture medium was exchanged for medium containing the different compounds. The culture medium of the untreated cells was refreshed at the same time point. Primary fibroblasts were kept on the same medium for 7 days, HEK cells for 4 days and transformed/immortalized fibroblasts for 24 h or 7 days. Transformed/immortalized fibroblasts that were cultured for 7 days were split 1:5 after 4 days and fresh medium containing the compound was added.

### Immunofluorescence Microscopy Assays

For immunofluorescence microscopy, cells were cultured on cover slips and incubated with the different compounds for the indicated times, followed by fixation and permeabilization with phosphate-buffered saline (PBS, Fresenius Kabi GmbH, Austria) solution containing 2% paraformaldehyde (Merck 8.18715.0100) and 0.1% Triton X-100 (BIO RAD 161-0407) for 20 min. After quenching free aldehyde groups with 100 mM ammonium chloride (Merck 1.01145.1000; 10 min incubation), the cells were consecutively incubated with primary and secondary antibodies and streptavidin–fluorescein isothiocyanate (FITC) complex (DAKO F 422) diluted in 1% bovine serum albumin (BSA) in PBS for 45 min. The glass slides were fixed on objective slides with the mounting medium Vectashield H1000 (Brunswick) or – for staining of nuclei – with ProLong^TM^ Diamond Antifade Mountant with DAPI (Thermo Fisher, Waltham, MA, United States). We used primary antibodies against catalase (mouse monoclonal, Mab 17E10, own generation (Wiemer et al., 1992)) and ABCD3 (PMP70) (rabbit polyclonal, Zymogen, 718300, diluted 1:500), and, as secondary antibodies, Alexa Fluor 555 Rabbit IgG (Z25305, ThermoFisher, diluted 1:500) or streptavidin–FITC complex (Bender Medsystems, diluted 1:200).

### Imaging and Image Processing

Live cells were imaged with a Leica TCS SP8 filter-free spectral confocal microscope using a 63× oil immersion objective and the Leica LAS Lite Software. We used excitation wavelengths of 489 nm for GFP signal, 495 nm for FITC signal and 555 nm for AF555 signal, and emission spectra were obtained at 505–550 nm, 499–553 nm, or 597–620 nm, respectively (HyD SMD detectors). Acquirement settings (e.g., the intensity of white light laser) were optimized for each experiment, and identical settings were used for all images throughout one experiment, allowing comparison between the various incubation conditions. Images of cells per condition were acquired in two or three different areas of the glass slides with a comparable confluency of cells (60–90%). Brightness and contrast were only adjusted for representative images demonstrating the quantified results for presentation purposes using identical parameters.

Image processing was performed off-line using the ImageJ software (version 150.i). For determining the density of punctated structures (i.e., the number of structures per cell area), five cells per image were manually outlined prior to the automated quantification process. The area size of each ‘region of interest’ as well as the number of contained punctated structures was measured, and the punctated structures per area size determined (‘density’). For quantification of the punctated structures, the red (for ABCD3 quantification) or green channel (for GFP quantification) was selected, the background noise removed (‘Subtract Background’) and appropriate thresholds applied further remove cytosolic background signals (‘Maximal Entropy’ filter). The ‘Watershed’ tool was used to separate overlapping punctated signals. Finally, the numbers of punctated structures were quantified (‘Analyze particles’ tool, size 2-infinity, circularity 0.5–1.0).

Due to the differential GFP signal strengths in the tr/immPEX1-G843D + GFP-PTS1 cells, the number of GFP punctated structures had to be manually corrected. We calculated the average density of structures determined in all cells, including cells with only cytosolic signal. In addition, we determined the proportion of cells displaying punctated structures and the average density of structures in these cells.

### Immunoblot Analysis

Immunoblot analysis was performed as previously described (Berendse et al., 2013) using cell homogenates prepared by resuspending cell pellets in lysis buffer [PBS with 0,25% Triton X-100 and protease inhibitor cocktail (Roche, Mannheim, Germany)] followed by sonication on ice water (8W, 40J). Proteins were separated by SDS-polyacrylamide gel electrophoresis (NuPAGE 10% Bis-Tris, Thermo Fisher, Waltham, MA, United States) using MES-SDS running buffer (NuPAGE, Thermo Fisher, Waltham, MA, United States) and subsequently transferred onto a nitrocellulose membrane using semidry blotting. As primary antibodies we used rabbit polyclonal antibodies against thiolase (Atlas antibodies HPA007244; 1:2000 dilution), ABCD3 (PMP70) (Thermo Fisher PA1-650; 1:1000 dilution), ACBD5 (Sigma HPA012145; 1:500 dilution), ACOX1 (Proteintech cat no 10957-1-AP; 1:1500 dilution), LC3B (Abcam ab48394; 1:1500 dilution), SQSTM1/P62 (BD Transduction lab; 1:2000 dilution). For loading control, we used a mouse monoclonal antibody against α-tubulin (Sigma T9026; 1:2000 dilution) or against b-actin (Sigma; 1:10000 dilution). For visualization, we used secondary antibodies IRDye 800 CW goat anti-rabbit (1:10.000 dilution) and IRDye 680CW donkey anti-mouse (1:10.000 dilution). The Odyssey Infrared Imaging System software (LI-COR Biosciences, Lincoln, NE, United States) was used for western blot quantification.

### Biochemical Assays

The β-oxidation rates of cerotic acid (C26:0) and pristanic acid were measured in cultured cells using radioactive labeled substrate as described (Wanders et al., 1995). C26:0-lysoPC and C26:0-carnitine were measured in cells as described (van de Beek et al., 2016). Peroxisome-dependent *de novo* ether phospholipid synthesis was assessed by measuring the *de novo* synthesis of the ether phospholipid PC-(O-37:4) following incubation of cells with 40 μM 1-heptadecanol (C17:0-OH) in standard culture medium for 24 h at 37°C. After trypsinization, cells were washed twice with PBS. After centrifugation, cell pellets were resuspended in 0.9% NaCl solution and sonicated on ice for 20 s at 7.5 W using a tip sonicator. Protein concentration was measured using the bicinchoninic acid assay (Smith et al., 1985). Analysis of the phospholipids was done essentially as described by Herzog et al. (2016). PC-(28:0) was used as internal standard for PC-(O-37:4). *De novo* synthesis of PC-(O-37:4) is expressed as PC-(O-37:4)/PC-(28:0)/mg protein.

### Generation of PEX1-G843D HEK 293 Cells by CRISPR-CAS9

The CRISP-Cas9 genome editing technology (Ran et al., 2013) was used to introduce the c.2528G>A (p.G843D) missense mutation in *PEX1* of HEK 293 Flp-In cells. To this end, two complementary oligonucleotides coding for a guide RNA upstream of a proto-spacer adjacent motif (PAM) site in exon 15 of the *PEX1* gene and a single-stranded oligo donor (ssODN) repair template were designed using the online CRISPR design tool^1^. The two oligonucleotides (oligo 1: PEX1-2493-fw CACCG CCAGGTCTCTAGGTTTATGC and PEX1-2512-rev aaac GCATAAACCTAGAGACCTGG C – oligo 2: PEX1-2511-fw CACCG CACCAATCTTGTCCCAACCC and PEX1-2530-rev aaac GGGTTGGGACAAGATTGGTG C) were annealed and subsequently cloned into the vector pspCas9n(BB)-2A-GFP (Addgene 48140), followed by Sanger sequencing of the insert to confirm the correct sequence. HEK 293 Flp-In cells were transfected using jetPRIME (Polyplus transfection, New York, NY, United States) with 1.5 μg plasmid and 0.6 μg ssODN repair template containing the PEX1-G843D missense mutation (CAGGTAACTGGATAGTATCCATGAGTATCTGTCTAACTT CATGTAACCCAtCAATCTTGTCCCAACCCAGATCTCTAGG TTTATGCAGATTGACACTTCG). After 2 days, GFP-positive cells, indicating successful transfection with pspCas9n(BB)-2A-GFP, were sorted into wells of a 96-well plate using fluorescence-activated cell sorting (FACS) flow cytometry (S800H Cell Sorter, Sony). After approximately 3 weeks, genomic DNA was isolated from cell pellets using Phire Animal Tissue Direct PCR Kit (Thermo Fischer, Waltham, MA, United States). Exon 15 of the *PEX1* gene was PCR amplified using specific primers for *PEX1* (available upon request) tagged with a -21M13 (5′ TGTAAAACGACGGCCAGT-3′) sequence or M13rev (5′-CAGGAAACAGCTATGACC-3′) sequence, respectively. Sequence analysis was performed using the Big DyeTM Terminator v.3.1 Cycle Sequencing Kit (Applied Biosystems, Foster City, CA, United States) on an ABI 3730 sequencer (Applied Biosystems, Foster City, CA, United States) using -21M13 or M13rev primers.

### Genetic Depletion of ATG5 and NBR1 in Primary PEX1-G843D and Control Fibroblasts

To generate primary PEX1-G843D and control fibroblasts that stably express shRNAs against ATG5 or NBR1, we used specific NBR1 and ATG5 shRNA-containing plasmids from the MISSION^®^ TRC library (Sigma-Aldrich), and, as a control, the non-target scrambled shRNA SHC002 (Sigma-Aldirch). Viral particles containing the different shRNAs were generated in HEK 293 cells co-transfected with each of the shRNA plasmids and the lentiviral packaging plasmids pMD2G, pMDL/RRE, pRSV/REV using jetPRIME^®^ (Polyplus Transfection) according to the manufacturer’s protocol. 48 h after transfection, viral supernatants were collected and used for infection of the human primary fibroblasts. 24 h after infection, the human primary fibroblasts were selected with puromycin (Sigma-Aldrich). To test the knockdown efficiency of NBR1, total RNA was isolated from puromycin-selected human primary fibroblasts using trizol extraction (Sigma-Aldrich) followed by cDNA synthesis using the QuantiTect Reverse Transcription Kit (QIAGEN). Quantitative PCR was preformed using LightCycler 480 SYBR Green I Master (Roche) on the LightCycler 480 (Roche). For data analysis, LightCycler 480 software release 1.5.0 (Roche) and the LinRegPCR program version 2014.5 (Ramakers et al., 2003). were used. Expression levels were corrected by the geometric mean of PPIB and 36B4. To test the knockdown efficiency of ATG5, we performed immunoblot analysis as described above using an ATG5-specific primary antibody (Proteintech; 1:1000 dilution) and IRDye 800CW goat anti-rabbit antibodies (LI-COR Biosciences).

## Data Availability Statement

The original contributions presented in the study are included in the article/supplementary material, further inquiries can be directed to the corresponding author/s.

## Author Contributions

FK, KF, RO, JK, and DG: experiments. FK, KF, RO, JK, SF, RW, and HW: interpretation of data. FK, KF, and HW: writing of original manuscript. FK, KF, RO, JK, SF, RW, and HW: revision and approval of manuscript. All authors contributed to the article and approved the submitted version.

## Conflict of Interest

The authors declare that the research was conducted in the absence of any commercial or financial relationships that could be construed as a potential conflict of interest.

## Footnotes

1 http://crispr.mit.edu/
